# An optomechanical MEMS geophone with a 2.5 ng/Hz^1/2^ noise floor for oil/gas exploration

**DOI:** 10.1038/s41378-024-00802-5

**Published:** 2024-11-26

**Authors:** Shimin Jiao, Ziqiang Qu, Xujin Ma, Hao Ouyang, Wen Xiong, Shaolin Zhang, Qiu Wang, Huafeng Liu

**Affiliations:** 1 PGMF and School of Physics, Huazhong University of Science and Technology, Wuhan, 430074 China; 2 Optics Valley Laboratory, Hubei, 430074 China

**Keywords:** Optical sensors, Micro-optics

## Abstract

High-precision geophones play crucial roles in terrestrial applications such as oil and gas exploration as well as seismic monitoring. The development of optomechanical precision measurements provides a new design method for geophones, offering higher sensitivity and smaller dimensions compared to traditional geophones. In this work, we introduce an optomechanical microelectromechanical system (MEMS) geophone based on a plano-concave Fabry‒Perot (F–P) microcavity, which has a high sensitivity of 146 V/g. The F‒P microcavity consists of a movable mirror on the sensing element and a fixed hemispherical micromirror fabricated from silicon-on-insulator (SOI) and monocrystalline silicon wafers, respectively. The experimental results show that the geophone has a low noise floor of 2.5 ng/Hz^1/2^ (with a displacement noise floor of 6.2 fm/Hz^1/2^) within the frequency range of 100~200 Hz, a broad bandwidth of 500 Hz (–3 dB), and a measurement range of ±4 mg. To mitigate common-mode noise originating from the laser source and environmental factors such as temperature and air fluctuations, a balanced detection method is employed. This method substantially reduces the noise floor, nearly reaching the thermal noise limit (2.5 ng/Hz^1/2^). Furthermore, a compactly packaged optomechanical MEMS geophone with a diameter of 40 mm is demonstrated. The high performance and robust features hold great potential for applications in oil and gas exploration.

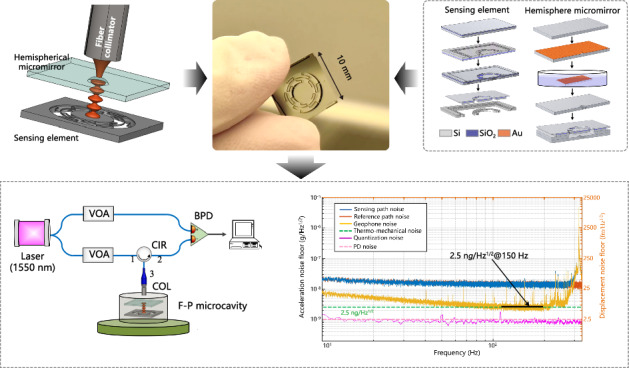

## Introduction

In oil and gas exploration, the seismic reflection method is a common practice and is utilized to delineate the subsurface structure of the Earth through the artificial generation of seismic waves that are detected by geophones and seismometers^1,2^. Especially in underground environments, the temperature increases from 3 °C to 5 °C every 100 m. This poses a significant challenge to the robustness and interference resistance of seismic sensors. Geophones are much less expensive than seismometers, therefore, geophones are more likely to be used in arrays for large-area seismic exploration. In addition, geophones also serve as essential sensors for earthquake monitoring^3^, vibration measurement^4^, and structural health monitoring^5^. Functioning essentially as accelerometers, geophones comprise a framework, springs, a proof mass, and a readout system. Conventional geophones are specifically of the moving-coil electromagnetic type and respond to ground motion above their natural frequency^6,7^. Therefore, conventional geophones operate as velocity meters with a flat velocity frequency response and bandpass characteristics, leading to poor performance in low-frequency applications. In addition, the assembly of conventional moving-coil geophones involves multiple large components, such as frames, coils, inertia masses, and springs, resulting in products that are quite expensive, bulky, and lack repeatability in their measurement performance^8^.

With advancements in microfabrication technology, there has been a notable ability to reduce the size and weight of seismic sensors. Emerging microelectromechanical system (MEMS)-based geophones offer a flat acceleration frequency response and low-pass characteristics, covering a broad frequency band from DC to 500 Hz^9^. Therefore, MEMS geophones tend to have better low-frequency responses and smaller sizes than conventional geophones. At present, a series of technologies, such as bulk silicon fabrication^10^, capacitor fringe effects^11^, and differential measurements^12^, have been developed in capacitive MEMS accelerometers to achieve ng-level acceleration measurements. However, limitations arise, from parasitic capacitance and susceptibility to electromagnetic interference, restricting the capacitive detection method to subpm displacement resolution and impeding its application in precision measurement fields^13,14^. Higher-precision displacement measurement methods are achieved by using optical shadows^15,16^, optical grating diffraction^17,18^, and cavity interferometry^19^. Compared with traditional accelerometers, which are based on capacitive, piezoelectric, and piezoresistive principles, optical accelerometers have the advantages of greater sensitivity, smaller dimensions, and intrinsic immunity to electromagnetic noise^20,21^.

More recently, the optomechanical MEMS system, which integrates a mechanical oscillator with an optical cavity, has emerged as a promising research area for high-precision measurements of small forces, masses, displacements, and accelerations^22–24^. Numerous innovative designs of optomechanical accelerometers have been studied for precision measurements ranging from macroscale gravitational wave detectors to microscale oscillators.

The laser interferometer gravitational wave observatory comprises two widely separated 4 km laser interferometers that introduce an optomechanical cooling technique. This method has potential for approaching the quantum ground state of a kilogram-scale oscillator. The oscillator operates within a factor of 10 of the standard quantum limit, achieving a displacement measurement of 10^−18^ m and an effective temperature of 1.4 μK. Such ultrahigh-precision displacement measurements form the foundation of gravitational wave detection^25^.

Painter et al. demonstrated a microchip optomechanical accelerometer utilizing a photonic-crystal nanocavity monolithically integrated with a nanotethered proof mass that exhibits a high mechanical *Q* factor of 10^6^. This device achieves an acceleration noise floor of 10 μg/Hz^1/2^ and a broad bandwidth exceeding 20 kHz, with an equivalent displacement noise as low as the fm/Hz^1/2^ level. Notably, a strong thermo-optomechanical backaction mechanism is observed, effectively damping and cooling the thermal motion of the proof mass^26^.

In a separate study, the National Institute of Standards and Technology reported an optomechanical accelerometer utilizing a plano-concave F–P cavity, achieving a thermal noise limit of resolution of 93 ng/Hz^1/2^ over a frequency range greater than 13 kHz. The F‒P cavity with an optical finesse of 5430 demonstrates an ultrahigh sensitivity of 6000 V/g. The optomechanical sensing method allows direct acceleration measurement in terms of the laser wavelength, making it possible for sensors to calibrate internally and serve as intrinsic standards^27^.

For accelerometers based on optomechanical systems, an optical microcavity is used to resonantly enhance the readout of mechanical motion to achieve remarkable fm/Hz^1/2^ level displacement resolution. Typically, the fundamental frequency of a mechanical oscillator is generally designed within the range of 10–100 kHz, resulting in acceleration noise at the subμg/Hz^1/2^ level^28^. Hence, by appropriately designing the fundamental frequency of the mechanical oscillator, a high-precision accelerometer at the ng/Hz^1/2^ level can be realized. The theory of cavity optomechanics enables the accelerometer to achieve a delicate balance between low noise and broad bandwidth. Furthermore, the combination of accelerometers and optomechanical systems offers the unique capability of controlling the sensor bandwidth via the optical spring effect^29,30^ and the effective temperature of thermomechanical motion by passive damping^31,32^.

In this work, we introduce a miniaturized optomechanical MEMS geophone with a noise floor of 2.5 ng/Hz^1/2^, a bandwidth of 500 Hz, and a measurement range of ±4 mg. This represents a significant enhancement in resolution and bandwidth compared with conventional and capacitive MEMS geophones. Additionally, we develop a microfabrication technology to integrate a plano-concave F‒P microcavity comprising a movable micromirror and a fixed hemispherical micromirror. Compared with traditional parallel F‒P cavities, the plano-concave characteristic enhances the displacement sensitivity by a factor of 4. Furthermore, to mitigate common-mode noise originating from the laser source and temperature/air fluctuations, we adopt the balanced differential detection method, which uses reference light and sensing light. The common-mode noise of the two light paths is suppressed through the data differential in the time domain. The testing method substantially reduces the noise floor from 15 ng/Hz^1/2^ to 2.5 ng/Hz^1/2^, thereby reaching the mechanical thermal noise limit. Moreover, the proposed geophone exhibits robust capabilities for effective sensing in harsh environments through the suppression of environmental noise. Detailed discussions on the structural designs, microfabrication processes, and testing methods of the optomechanical geophone are provided in the subsequent sections.

## Principle and fabrication

**Fig. 1:**
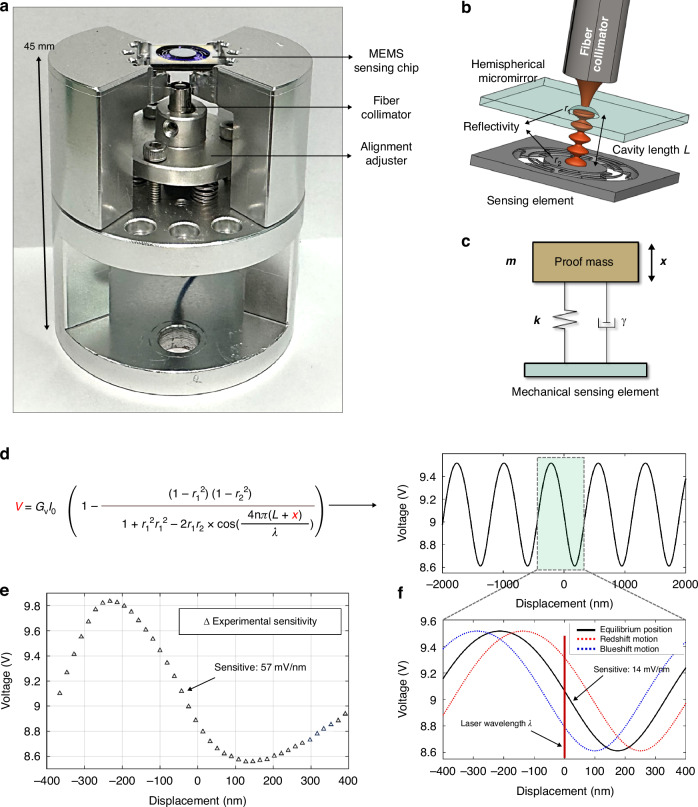
Concept of the optomechanical MEMS geophone. **a** Assembly drawing of the geophone provides flexible alignment between the fiber light and F‒P microcavity, including a MEMS sensing chip, a fiber collimator, and an alignment adjuster. **b** Diagram of the optomechanical F‒P microcavity comprising a fixed hemispherical micromirror and a movable mirror on the sensing element. **c** Ideal second-order spring-mass system. **d** Acceleration detection principle based on F‒P cavity multibeam interference. **e** Experimental displacement sensitivity of the geophone tested via a calibration experiment, as shown in Fig. 4b. **f** Theoretical displacement sensitivity calculated via Eq. (3)

The optomechanical geophone with a diameter of 40 mm, a height of 45 mm, and a weight of 75 grams is composed of three primary components: a MEMS sensing chip, a fiber collimator, and an alignment adjuster. The alignment adjuster ensures the precise collimation of Gaussian light from the fiber into the microcavity at normal incidence (Fig. 1a). The design of the plano-concave F‒P microcavity features a movable mirror on the sensing element and a fixed hemispherical micromirror (Fig. 1b). The hemispherical micromirror aims to lessen the demand for precise parallelism in the F‒P cavity through its rotational symmetry while simultaneously enhancing sensitivity. The sensing element comprises a proof mass and suspended beams that enable the out-of-plane motion of the proof mass. The mode frequency of this motion can be theoretically calculated via Eq. (1)^33^:

1 $${{f}}_{0}=\frac{1}{2\uppi}\sqrt{\frac{{nEb}{{h}}^{3}}{{m}{{l}}^{3}}}$$

where *f*_0_ is the fundamental frequency; *n* is the number of beams; *E* is the Young’s modulus of the silicon material (170 GPa); *b* (width), *h* (thickness), and *l* (length) denote the dimensions of the beam (30 μm, 100 μm, and 8000 μm, respectively); and $m$ is the weight of the proof mass (17.5 mg). Substituting these values into Eq. (1), the calculated fundamental frequency of the sensing element is *f*_0_ = 294 Hz.

The principle of the optomechanical geophone is divided into the mechanical conversion of acceleration into displacement by the sensing element and optical interference displacement measurement by the F‒P cavity. The sensing element is conceptualized as an ideal second-order response system characterized by its fundamental frequency ${\omega }_{0}$ (Fig. 1c). The frequency-dependent mechanical response can be mathematically expressed as

2 $$a(\omega )=\left({\omega }_{0}^{2}-{\omega }^{2}-i\frac{\omega {\omega }_{0}}{Q}\right)\times x(\omega )$$

where $a\,(\omega )$ is the external acceleration; ${\omega }_{0}\,=\sqrt{k/m}=\,$2$\pi$*f*_0_; $k$ is the spring stiffness; *Q* is the quality factor of the sensing element; and $x\,(\omega )$ is the displacement of the proof mass. When the frequency of the detected external acceleration is substantially lower than its fundamental frequency $(\omega \ll {\omega }_{0})$, the relationship between the acceleration *a* and the displacement *x* can be simplified as a linear gain *a* = *ω*_0_^2^*x*. Considering the displacement detection within the F‒P cavity, the input acceleration generates a perturbation in the cavity length, thereby altering the phase of the reflected light and subsequently causing a variation in the interference light intensity. The generic reflected intensity of multibeam interference *I*_*R*_ can be expressed as

3 $${I}_{R}={I}_{0}\times \left(1-\frac{(1-{{r}_{1}}^{2})(1-{{r}_{2}}^{2})}{1+{r}_{1}^{2}{r}_{1}^{2}-2{r}_{1}{r}_{2}\times \cos \varphi }\right)$$

where ${I}_{0}$ is the optical power incident upon the F–P cavity; ${r}_{\mathrm{1,2}}=\sqrt{{R}_{\mathrm{1,2}}}$ is the square root of the reflectivity (*R*_1,2_) of the hemispherical micromirror and the sensing element micromirror; and the phase term *φ* = 4*n*π(*L* + *x*)/*λ* contains the displacement *x*, the cavity length *L* (850 μm) and the laser wavelength $\lambda$ (1550 nm). The reflectivity of the hemispherical mirror is determined by the monocrystalline silicon (*R*_1_ = 0.04), while the surface of the sensing element is coated with a gold film to increase its reflectivity (*R*_2_ = 0.9).

When the optomechanical geophone is working, the incident light passes through the hemispherical mirror and is reflected by the sensing element. The laser wavelength *λ* is locked to the sideband of the resonant wavelength (~1550 nm) (Fig. 1d). Within the linear sensitivity region, the acceleration-induced displacement of the sensing element changes the cavity length, consequently causing a variation in the output optical power. Considering the incident light power *I*_0_ (1.2 mW) and the photodetector (PD) gain g_*v*_ (10^4^ V/W), the theoretical displacement sensitivities are calculated to pass through 14 mV/nm. However, through experimental calibration, the achieved sensitivity is 57 mV/nm. This discrepancy between the theoretical and experimental values indicates that the hemispherical microcavity enhances the sensitivity by a factor of 4.

## Device fabrication

**Fig. 2:**
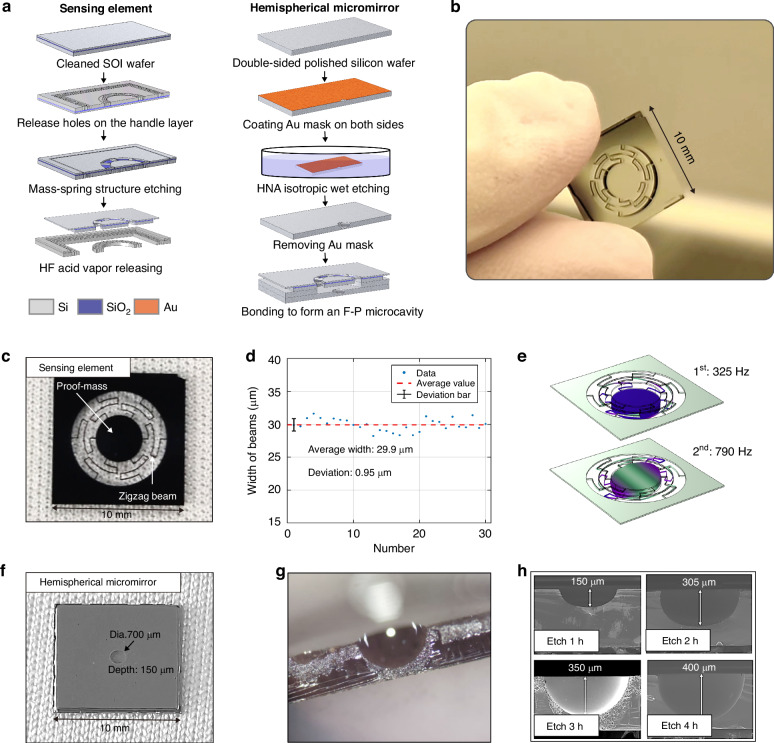
MEMS processes for the optomechanical geophone. **a** MEMS process for the sensing element and hemispherical micromirror. **b** As-fabricated optomechanical F‒P microcavity comprising a fixed hemispherical micromirror and a movable mirror on the sensing element. **c** Diagram of the sensing element with a size of 10 mm. **d** Deviation of the beams of the sensing element in the MEMS process. **e** Finite element simulation of the sensing element with the first mode at 325 Hz and the second mode at 790 Hz. **f** Diagram of the hemispherical micromirror with a diameter of 700 μm and a depth of 350 μm. **g** Optical microscopy image of a hemispherical micromirror. **h** SEM images of the hemispherical micromirror at various etching times

Since the F‒P microcavity comprises both a mechanical sensing element and a hemispherical micromirror, the sensing mode of the mechanical element requires out-of-plane motion capability. Considering the transparent properties of silicon materials for infrared light, we utilize silicon-on-insulator (SOI) and monocrystalline silicon wafers to fabricate the plano-concave F‒P microcavity via a bulk silicon process. The as-fabricated optomechanical structures have dimensions of 10 mm, and the MEMS microfabrication methodology employed in this work is detailed in the following sections (Fig. 2a, b).

## Sensing element

The sensing element consists of a frame, a proof mass, and six zigzag beams employed to increase the effective beam length and enhance the *Q* factor by effectively reducing the energy associated with the second mode^34^ (Fig. 2c). The sensing element is fabricated on a double-sided polished SOI wafer, which consists of a 100 μm thick device layer for processing zigzag beams, a 500 μm handle layer for the proof mass, and a 2 μm thick buried oxide layer in the middle. First, a series of release holes are etched into the handle layer via lithography and deep reactive ion etching (DRIE) for subsequent release of the suspended beams. Then, the beams (8000 μm in length, 30 μm in width, and 100 μm in thickness) and the proof mass (with a diameter of 4000 μm and thickness of 600 μm) are etched in the device layer via lithography and DRIE. Finally, the sensing element is released by hydrofluoric acid vapor etching, which eliminates the intermediate buried oxide layer.

The process deviation resulting from overetching during the DRIE process is less than 1 μm (Fig. 2e). Although the designed beams are intended to have a width of 30 μm, the actual width across the wafer varies between 29.15 μm and 30.99 μm, and the corresponding fundamental frequency spans from 315 Hz to 375 Hz. Consequently, the beam of the measured sensing element has a width of 29.2 μm, and its fundamental frequency of 317 Hz falls within the margin of deviation, aligning closely with the intended design, despite slight deviations. The finite element analysis reveals simulated frequencies for the first and second modes of the sensing element of 325 Hz and 790 Hz, respectively (Fig. 2e).

## Hemispherical micromirror

The hemispherical micromirror is fabricated on a 500 μm thick silicon wafer through isotropic wet etching in a mixed solution of hydrofluoric, nitric, and acetic acids (HNA, 9:75:30 ratio). First, to protect the double-sided polished silicon wafer from hydrofluoric acid, which is very corrosive, a 200 nm thick gold coating is applied to both sides, serving as the hard mask layer for wet etching^35^. Simultaneously, a lift-off process is employed to fabricate a series of 300 μm diameter circles on the gold mask, designated wet etching windows. The wafer is subsequently subjected to etching in the mixed solution and is stirred with a magnetic stir bar at 60 °C. The resulting hemispherical micromirror is attained by removing the gold mask and cleaning the wafer. Finally, the sensing element and the hemispherical micromirror are bonded via a 500 μm thick hollow silicon partition, which provides sufficient space between the moving-proof mass and the micromirror.

The selected parameters for the hemispherical micromirror include a depth of 350 μm and a diameter of 700 μm with a deviation of ±33 μm, facilitating mode matching within the plano-concave F‒P microcavity (Fig. 2f)^36^. The geometric parameters of the hemispherical micromirror exhibit proportional increases with etching duration, as observed through optical microscopy and scanning electron microscopy (SEM) (Fig. 2g, h).

## Results

### Setup

**Fig. 3:**
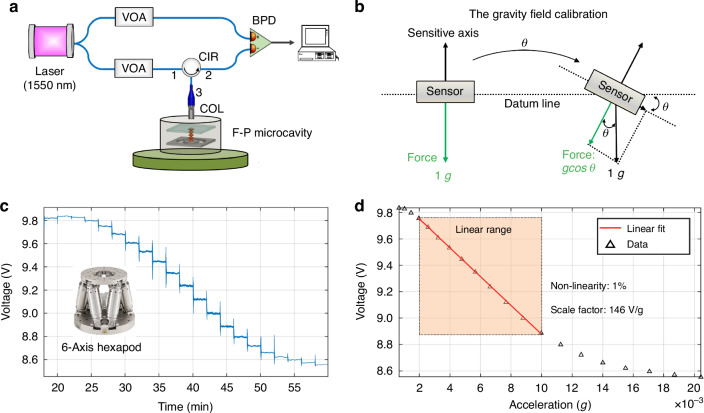
Calibration experiment on the optomechanical MEMS geophone. **a** Diagram of the optical readout method used to characterize the performance of the optomechanical geophone. A balanced difference method is used, which consists of a reference path and a sensing path, and two VOAs are used to adjust the optical power of the two paths. The sensing core is mounted on a PZT shaker. **b** Gravity field calibration based on a 6-axis hexapod; the sensitive axis is installed perpendicular to the table surface, and the displacement generated by gravity varies according to g*cosθ*. **c** Time-domain data of gravity step calibration, with the tilting range set from 0 to 8 degrees and a step of 0.2 degrees. **d** Fitting of the gravity step calibration data showing that the scale factor is 146 V/g with a nonlinearity of 1%

The optical readout system uses the balanced detection method, which can effectively suppress optical noise (Fig. 3a). The light emitted by the 1550 nm laser is divided into two paths, the sensing path, and the reference path, via a 1 × 2 fiber coupler. To ensure uniform intensity across both paths, two variable optical attenuators are employed. The sensing light passes through the fiber circulator (CIR) to reach the F‒P microcavity. Then, the interference light from the microcavity returns to the CIR and eventually reaches the balanced PD (Thorlabs: PDB435C-AC) with a 5 Hz high-pass filter. Simultaneously, the reference light is collected directly from the PDB. Additionally, the MEMS chip is mounted on the PZT shaker, enabling nm-scale displacement of the sensing element during dynamic testing. To evaluate the performance of the proposed optomechanical MEMS geophone, the following experiments are carried out.

### Static calibration

**Fig. 4:**
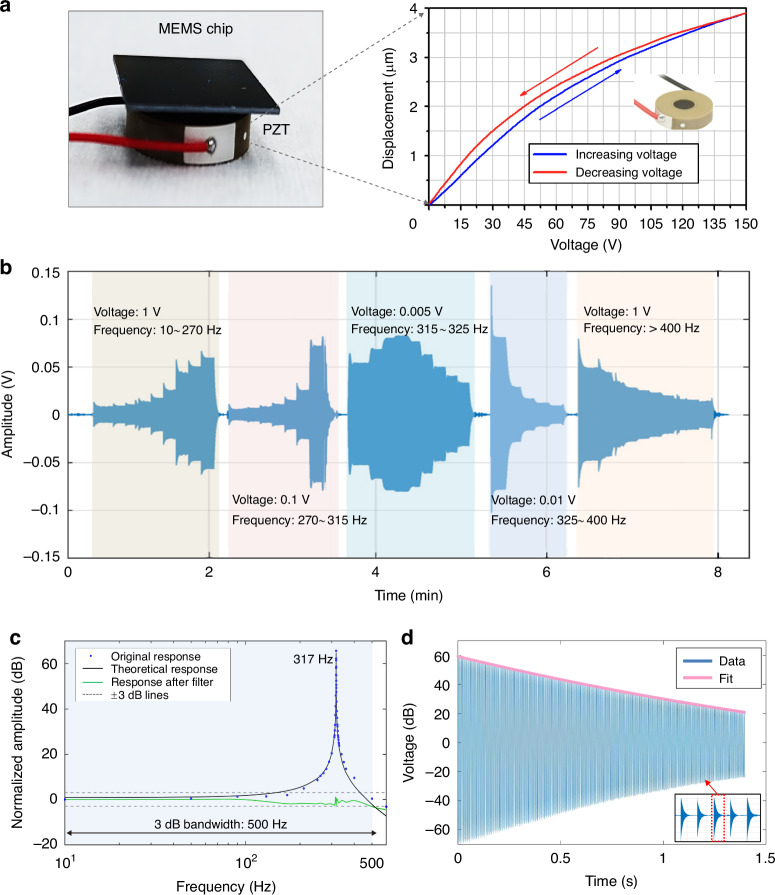
Dynamic experiment on the optomechanical MEMS geophone. **a** Test method based on a PZT shaker. The MEMS chip is mounted on the PZT shaker, which has a linear response in the low-voltage region. **b** Piecewise scanning frequency experiment; each segment adopts a different driving voltage for the PZT shaker, and the resonant peak can be completely swept out. **c** Testing and fitting of the amplitude-frequency response curve. **d** Ring-down response excited by the PZT shaker

The calibration experiment is crucial for determining the sensitivity between the input acceleration and output voltage. We utilize a gravity field calibration method, which involves applying a constant but small acceleration by tilting the dynamoelectric six-axis hexapod table. The sensitive axis of the geophone is vertically mounted on the surface of the table (Fig. 4a). The tilt range is set from 0 to 8 degrees, corresponding to a gravitational change following g*cosθ* (Fig. 3b).

For the time-domain data, each step is set at 0.2 degrees, with a 2-minute pause (Fig. 3c). The result shows a fitted scaling factor of 146 V/g with 1% nonlinearity (Fig. 3d). The measurement range can be calculated as ±4 mg. Owing to the asymmetric reflectivity of the mirrors at both ends of the F‒P microcavity, the calibration curve yields a peak-to-peak value of only 1.2 V.

### Dynamic response

Owing to the small measurement range and broad frequency bandwidth of the proposed geophone, commercial shaking tables are unsuitable for dynamic experiments. During dynamic testing, the MEMS chip is mounted on a PZT shaker (Thorlabs: PA44M3KW), which has a linear response within the low-voltage range of 0~15 V (Fig. 4a). This PZT shaker provides acceleration excitation with a fixed amplitude but sweeping frequency. However, the high *Q* factor of the sensing element severely distorts the amplitude-frequency response near the resonant frequency. To mitigate this, the sweep range is divided into 5 parts, each employing different driving voltages to counteract distortion (Fig. 4b).

A sweep-frequency experiment is performed to characterize the amplitude‒frequency response of the geophone (Fig. 4c). The original response data can be obtained by setting the sweep range from 10 to 600 Hz. The experimental data are then theoretically fitted by Eq. (2) with an *R*^2^ value of 0.9971, revealing a fundamental frequency of 317 Hz. On the basis of the response of the high *Q* factor, we design a digital compensation filter to suppress the interference of the resonant peak and broaden the bandwidth to 500 Hz (−3 dB) via the same method from the literature^37^. In addition, a series of ring-down curves is obtained by generating an excitation by generating a long-period step signal with the PZT shaker (Fig. 4d). The mechanical quality factor *Q* value of the sensing element is determined to be 3100 through data fitting.

Furthermore, the mechanical thermal noise *a*_*th*_ resulting from thermal Brownian motion can be described by Eq. (4):

4 $${a}_{{th}}=\sqrt{\frac{4{k}_{\text{B}}T{\omega }_{0}}{{mQ}}}$$

where *k*_B_ is the Boltzmann constant and *T* = 298.15 K is room temperature. By utilizing the measured values of ${\omega }_{0}$ and the *Q* factor, the mechanical thermal noise is calculated as *a*_*th*_ = 2.5 ng/Hz^1/2^.

### Noise performance

**Fig. 5:**
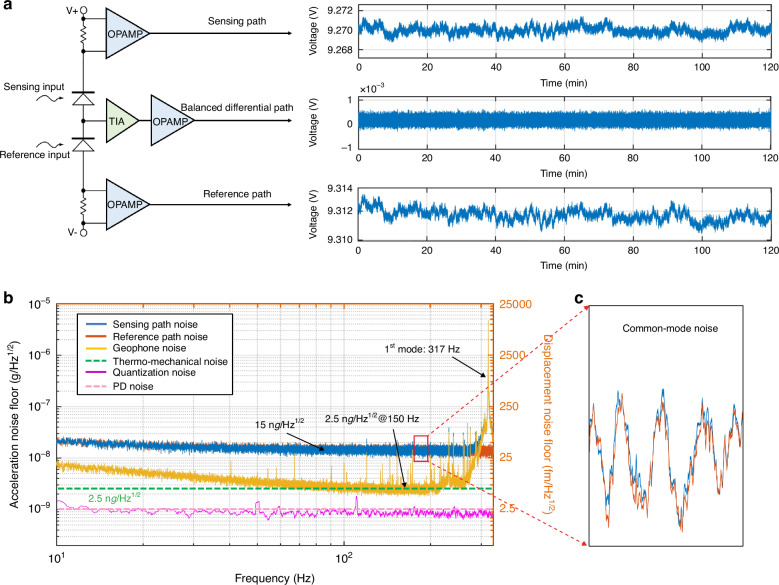
Noise performance experiment on the optomechanical MEMS geophone. **a** Optical readout based on the balanced difference method. The three-channel outputs include the sensing path, reference path, and balanced differential path. **b** Noise power spectral density of the three output channels. The blue curve represents the sensing path noise, the red curve represents the reference path noise, the yellow curve represents the sensing path noise, the purple curve represents the quantization noise, the green dotted line represents the mechanical thermal noise, and the pink dotted line represents the photodetector (PD) noise. **c** Common-mode noise between the sensing noise and reference noise

The resolution of the geophone can be quantified by noise-equivalent acceleration. The device is exposed to air and evaluated on a vibration isolation foundation. To suppress optical readout noise, a balanced detection method is employed (Fig. 5a). The reference signal and the sensing signal with the same power are collected and differentiated by two identical PDs. The three output channels simultaneously monitor the sensing path, the reference path, and the balanced differential path.

The acceleration noise power spectral density of the three output channels reveals that the geophone noise floor reaches 2.5 ng/Hz^1/2^ within the frequency range of 100~200 Hz (Fig. 5b). The largest dynamic range is calculated to be 124 dB, which is determined by the ratio of the maximum input acceleration (4 mg) to the noise floor^10^. Moreover, the equivalent displacement noise is calculated to be 6.2 fm/Hz^1/2^, which is derived from the mechanical response equation $a\,={\,\omega }_{0}^{2}x$. The noise peak at 317 Hz is caused by the mechanical response of the sensing element, which is consistent with the dynamic test results. The balanced detection method effectively suppresses common-mode noise originating from the laser source and temperature/air fluctuations (Fig. 5c). The geophone noise is reduced from 15 ng/Hz^1/2^ to 2.5 ng/Hz^1/2^, approaching the mechanical thermal noise limit (dashed curve), which is independent of frequency. In addition, the noise contribution from the interface electrons in the test system includes quantization noise introduced during data acquisition and PD noise. The quantization noise is measured at 0.8 ng/Hz^1/2^. The PD noise, as specified in the PD datasheet, is 1 ng/Hz^1/2^. Both are significantly lower than the mechanical thermal noise.

## Discussion and conclusion

Table 1 shows a performance comparison for the state-of-the-art geophones and accelerometers. Conventional moving-coil and MEMS geophones exhibit similar noise levels of 10 ng/Hz^1/2^, but MEMS geophones tend to have a better low-frequency response^38,39^. In terms of the noise floor, the MEMS capacitive accelerometer achieves the lowest noise floor of 0.25 ng/Hz^1/2^; however, it comes at the expense of bandwidth. Optical MEMS accelerometers can reach noise levels of ng/Hz^1/2^ but exhibit relatively narrow bandwidths. In contrast, compared with other MEMS geophones the proposed optomechanical MEMS geophone has a low noise floor of 2.5 ng/Hz^1/2^ and a broad bandwidth from DC to 500 Hz. Additionally, the proposed geophone exhibits robust capabilities for effective sensing in harsh environments through the suppression of environmental noise.

**Table 1:** Performance comparison for state-of-the-art geophones and accelerometer

Device	Noise floor	Bandwidth	Work mode	Dynamic range	Sensing unit dimensions
Electromagnetic moving-coil geophone^38^	10 ng/Hz^1/2^	15~280 Hz	Closed-loop	155 dB	50 mm
Optical moving-coil geophone^18^	3 ng/Hz^1/2^	0.1~100 Hz	Closed-loop	176 dB	40 mm
MEMS capacitive geophone^39^	12 ng/Hz^1/2^	DC~800 Hz	Closed-loop	130 dB	10 mm
MEMS capacitive accelerometer^14^	0.25 ng/Hz^1/2^	DC~40 Hz	Closed-loop	—	25 mm
MEMS optical grating accelerometer^17^	2 ng/Hz^1/2^	DC~15 Hz	Open-loop	95 dB	15 mm
MEMS F-P cavity accelerometer^10^	2.4 ng/Hz^1/2^	DC~80 Hz	Open-loop	110 dB	20 mm
This work	2.5 ng/Hz^1/2^	DC~500 Hz	Open-loop	124 dB	10 mm

In conclusion, we demonstrated a compactly packaged optomechanical MEMS geophone based on a plano-concave F–P microcavity, exhibiting a low noise floor of 2.5 ng/Hz^1/2^, a broad bandwidth of 500 Hz, and a measurement range of ±4 mg. The equivalent displacement noise reaches 6.2 fm/Hz^1/2^, demonstrating the potential of F–P cavity interferometry in precision measurements. The experimental results illustrate that common-mode noise originating from the laser source and temperature/air fluctuations can be suppressed from 15 ng/Hz^1/2^ to the mechanical thermal noise limit of 2.5 ng/Hz^1/2^ through the use of a balanced detection method. Therefore, the high performance and robust capabilities of the proposed geophone render it suitable for applications in oil and gas exploration.
